# User Experiences of an Internet-Based Stepped-Care Intervention for Individuals With Cancer and Concurrent Symptoms of Anxiety or Depression (the U-CARE AdultCan Trial): Qualitative Study

**DOI:** 10.2196/16604

**Published:** 2020-05-19

**Authors:** Helena Igelström, Anna Hauffman, Sven Alfonsson, Jonas Sjöström, Åsa Cajander, Birgitta Johansson

**Affiliations:** 1 Department of Neuroscience Uppsala University Uppsala Sweden; 2 Department of Immmunology, Genetics and Pathology Uppsala University Uppsala Sweden; 3 Department of Women’s and Children’s Health Uppsala University Uppsala Sweden; 4 Department of Clinical Neuroscience Karolinska Institutet Stockholm Sweden; 5 Department of Informatics and Media Uppsala University Uppsala Sweden; 6 Department of Information Technology Uppsala University Uppsala Sweden

**Keywords:** interactive web portal, stepped care, user experience, cancer, interviews

## Abstract

**Background:**

The internet-based stepped-care intervention iCAN-DO, used in the multicenter randomized controlled trial *AdultCan*, was developed for adult patients undergoing treatment for cancer and concurrently experiencing anxiety or depressive symptoms. iCAN-DO aimed to decrease symptoms of anxiety or depression. Step 1 comprises access to a library with psychoeducational material and a peer-support section, as well as the possibility to pose questions to a nurse. Step 2 of the intervention offers treatment consisting of internet-based cognitive behavioral therapy (iCBT) to participants still experiencing anxiety or depression at 1, 4, or 7 months after inclusion.

**Objective:**

The study aimed to explore user experiences of delivery, design, and structure of iCAN-DO from the perspective of people with cancer.

**Methods:**

We studied user experiences by interviewing 15 informants individually: 10 women with breast cancer (67%), 4 men with prostate cancer (27%), and 1 man with colorectal cancer (7%) with a mean age 58.9 years (SD 8.9). The interviews focused on informants' perceptions of ease of use and of system design and structure. Informants had been included in iCAN-DO for at least 7 months. They were purposefully selected based on activity in Step 1, participation in iCBT (ie, Step 2), gender, and diagnosis.

**Results:**

Of the 15 informants, 6 had been offered iCBT (40%). All informants used the internet on a daily basis, but 2 (13%) described themselves as very inexperienced computer users. The analysis revealed three subthemes, concerning how user experiences were affected by disease-specific factors and side effects (User experience in the context of cancer), technical problems (Technical struggles require patience and troubleshooting), and the structure and design of iCAN-DO (Appealing and usable, but rather simple).

**Conclusions:**

The results indicate that user experiences were affected by informants’ life situations, the technical aspects and the design of iCAN-DO, and informants’ preferences. The results have generated some developments feasible to launch during the ongoing study, but if iCAN-DO is to be used beyond research interest, a greater level of tailoring of information, features, and design may be needed to improve user experiences. The use of recurrent questionnaires during the treatment period may highlight an individual’s health, but also function as a motivator showing improvements over time.

## Introduction

Today, many people with cancer are cared for as outpatients during cancer treatment [1], leaving limited occasions for meeting health care personnel. People with cancer may experience multiple symptoms from the disease and treatment [2] and might need information and advice for self-care of these symptoms [3]. Many patients have questions, especially during ongoing treatment, and would need information and advice, but there is a risk that these questions remain unanswered and patients live with unmet needs [4-6].

One option is to have patients access information and support functions via the internet. Internet-based support can provide relevant quality-assured information with just-in-time access, and can be combined with packages for enhanced social support and behavior change [7]. One advantage with this is that people can use such support when they feel ready for it or have time to access a computer or mobile device.

Internet-based support for people with cancer could be a way of providing information and support that may improve self-management of symptoms and thereby relieve distress and unmet needs. Further, it seems that improved access to information via the internet can stimulate engagement and empowerment [8]. In *AdultCan* [9], which is a multicenter randomized controlled trial (RCT), an internet-based stepped-care intervention, iCAN-DO, has been evaluated regarding its effects on anxiety and depressive symptoms in people with breast, colorectal, or prostate cancer with these symptoms.

If iCAN-DO is effective in reducing symptoms, the step toward clinical implementation is not far ahead. However, before implementing internet-based support into clinical care, the experience must be carefully explored from the user perspective. Important user-experience aspects to explore may be ease of use, navigation in the system, and perceptions of the design and structure [10].

This paper aims at exploring user experiences of delivery, design, and structure of the internet-based stepped-care intervention iCAN-DO used in *AdultCan* from the perspective of individuals with cancer who have symptoms of anxiety or depression.

## Methods

### Design

This was a qualitative study with an inductive approach using data collected through semistructured individual interviews.

### Setting

#### The Uppsala University Psychosocial Care Program and the Web Portal

We recruited the informants in this study from *AdultCan* (ClinicalTrials.gov Identifier: NCT-01630681), an RCT within the Uppsala University Psychosocial Care Program (U-CARE) [11]. U-CARE is a strategic research venture supported by the Swedish government and has a multidisciplinary composition involving caring science, psychology, and information systems. All U-CARE research projects deliver interventions and collect patient-reported data via a web portal (ie, *the portal*) developed within the U-CARE program.

At the time of the start of *AdultCan* in 2013, end users could access the portal using a two-step verification: (1) via username and password and (2) with a code sent by SMS. Most of the participant views employed the Bootstrap framework.

#### The AdultCan Trial

*AdultCan* targeted individuals with breast, colorectal, or prostate cancer with symptoms of anxiety or depression. Screening regarding symptoms of anxiety and depression was performed using the Hospital Anxiety and Depression Scale (HADS) [12]; participants with a score of more than 7 on either of the two subscales were randomized to the iCAN-DO or standard care groups.

**Figure 1:**
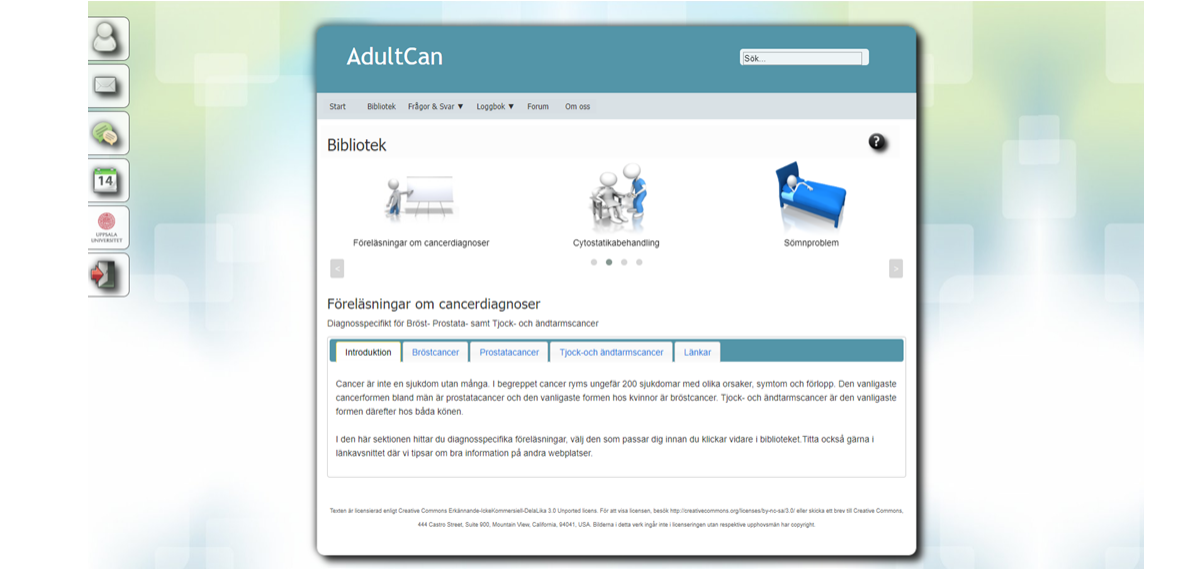
The start page of the portal (in Swedish).

**Figure 2:**
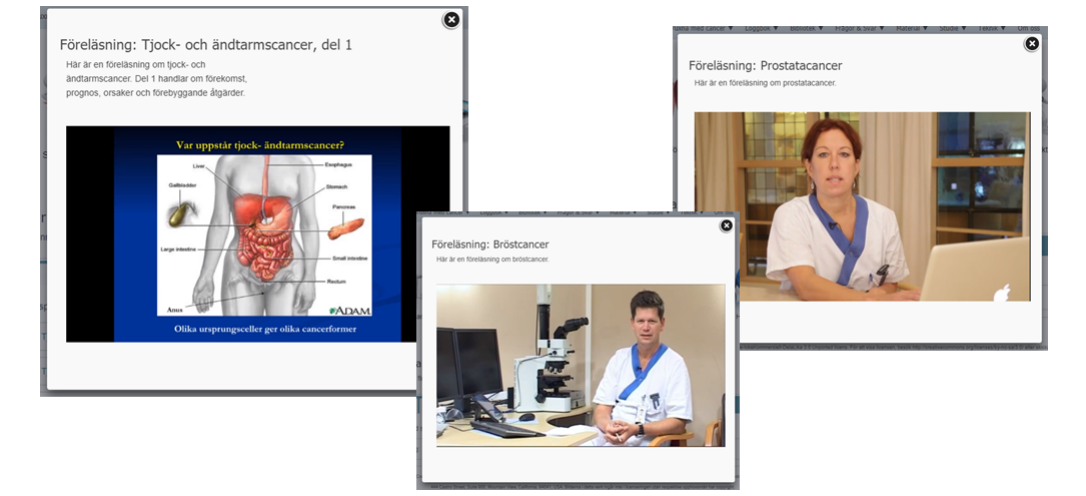
Diagnoses, with information on pathophysiology, prevalence, incidence, and prognosis (in Swedish).

**Figure 3:**
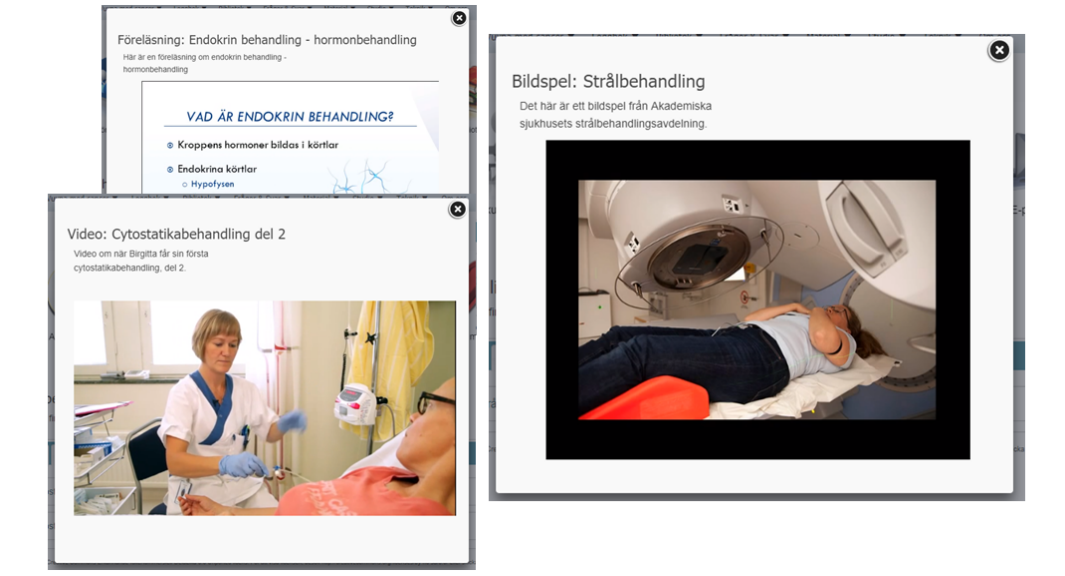
Oncological treatments: why and how (in Swedish).

**Figure 4:**
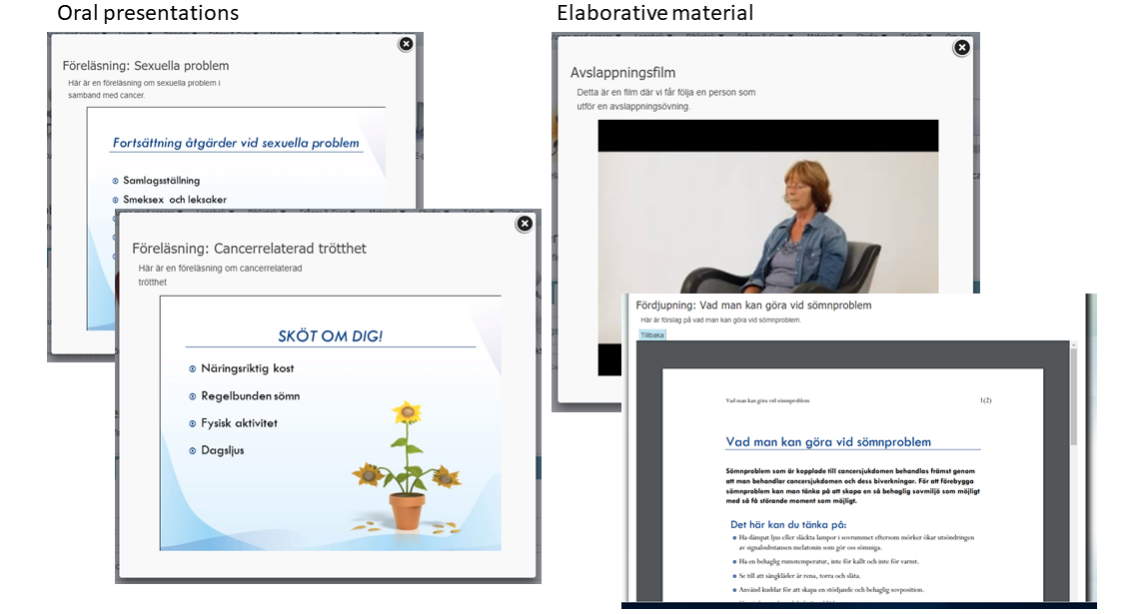
Symptoms and consequences of anxiety and depression as well as advice for self-care (in Swedish).

We developed the structure and content of iCAN-DO in collaboration with team members in clinical cancer care, system developers, and end users [13]. Step 1 of the intervention was accessible for 24 months and comprised a nurse-led interactive support program based on Orem’s Self-Care Deficit Theory and the concept of psychoeducation [14] and social cognitive theory [15]. A key part in this support program was an information library with materials and self-care advice concerning common problems surrounding cancer, such as pain, nausea, and sleeping problems (see Figures 1-4). Figure 1 illustrates the start page in the portal while the subsequent figures illustrate visual and written information on diagnosis (see Figure 2), treatment (see Figure 3), and advice for symptom management (see Figure 4).

**Figure 5:**
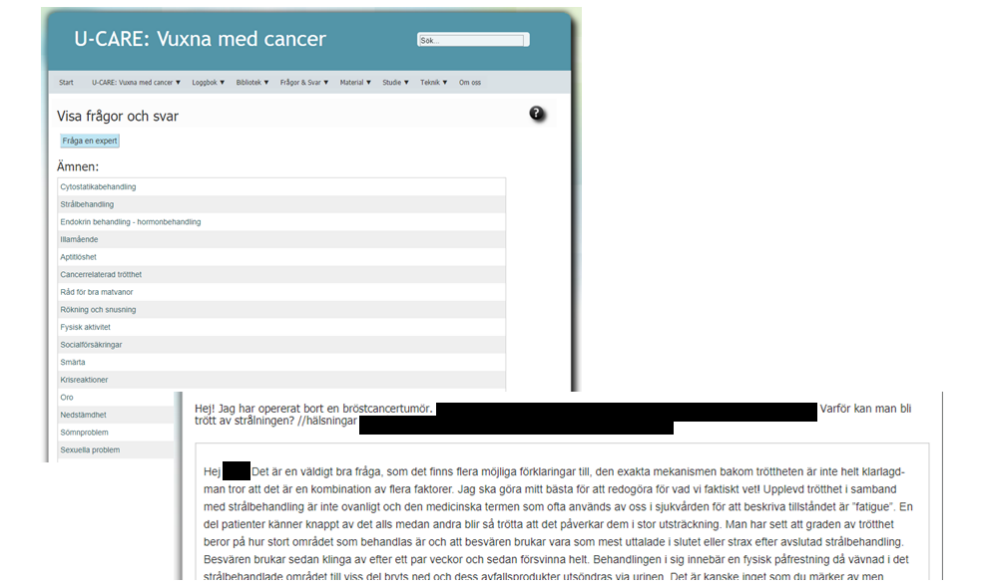
Frequently asked questions (FAQ), *Ask an expert*, and chat function (in Swedish).

Information was available in text, audiovisual presentations, slideshows, and video clips. All contents were visible to all users, though some parts of the information concerned a specific diagnosis. Step 1 also contained a forum, a chat function, and a frequently asked questions (FAQ) section, alongside the feature *Ask an expert*, where users could pose questions (see Figure 5). In order to strengthen the therapeutic alliance, the nurses (Step 1) and psychologist (Step 2) were presented with a photo and a brief description [16].

**Figure 6:**
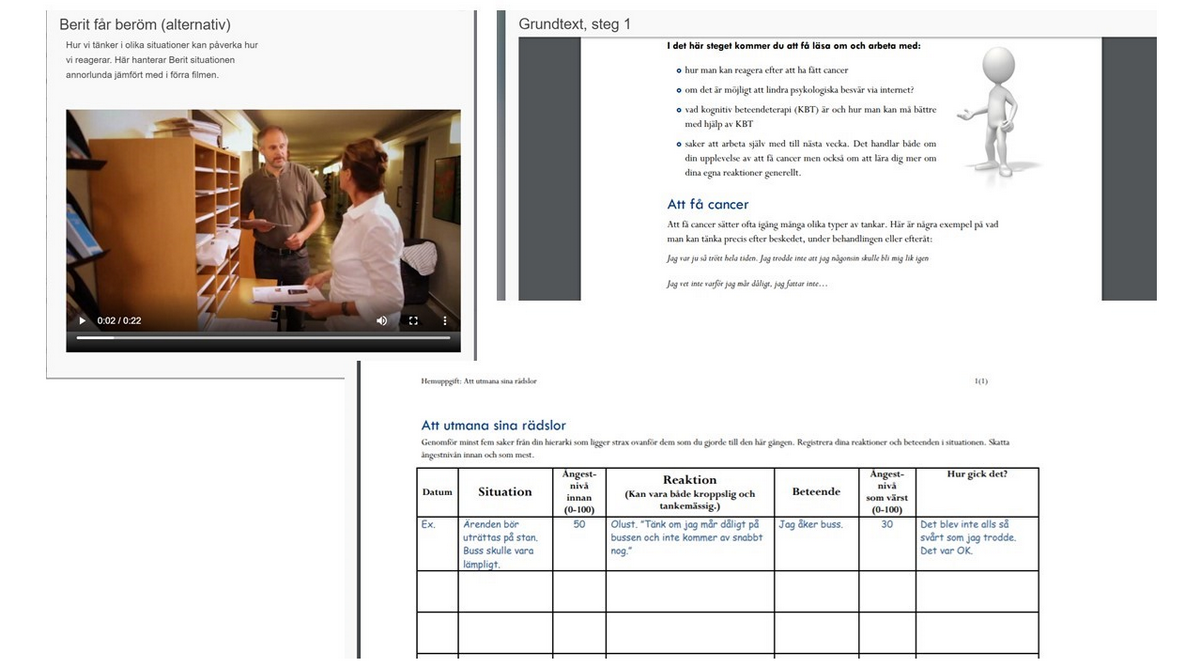
Examples of a video clip, text, and homework assignment in internet-based cognitive behavioral therapy (iCBT; in Swedish).

Step 2 in iCAN-DO constituted a 10-week internet-based cognitive behavioral therapy (iCBT) program [17] managed by a psychologist. iCBT was offered once and only to participants with prevailing symptoms of anxiety or depression (ie, score >7 on either HADS subscale) at 1, 4, or 7 months after inclusion. The treatment comprised 15 modules with written text, audiovisual presentations, and video clips covering a range of problem areas that were relevant for this population, including worry, fatigue, and depressed mood [18]. See Figure 6 for examples of a video clip, text, and homework assignment in iCBT.

All participants accepting iCBT were offered an introductory module, after which they were free to choose the most relevant modules to work with over 10 weeks. A psychologist guided the participants by monitoring their work, answering any questions, and delivering weekly feedback. All communication took place in the form of written messages via the portal.

### Informants

To be included, informants had to have had access to iCAN-DO for at least 7 months. In order to obtain variation among informants, a purposive selection was performed, mainly regarding activity in Step 1 and whether they participated in iCBT or not, but also regarding age, gender, and general online activity. A total of 20 individuals were approached, of whom 2 declined to participate and 3 were unreachable. In total, 15 informants were interviewed (see Table 1) in 2016 and 2017. All informants used the internet on a daily basis, but their general online activity varied. A total of 2 informants (13%) described themselves as very inexperienced computer users.

### Procedure

A letter was sent to potential informants with study information. They were informed that they would receive a phone call from research personnel within a couple of days asking about willingness to participate. If they did not want to receive this phone call, they could contact the person responsible for *AdultCan*; no one used that option. When a person consented to participate, a time and place for an interview were booked. All informants were treated with confidentiality and had time to consider their participation after getting written and verbal information. All provided signed informed consent. The study was approved by the regional committee for research ethics in Sweden, Uppsala county (Dnr 2012/003/9).

### Data Collection

#### Questionnaires and Log Data

Background information on informants was retrieved from self-reported questionnaires. All informants’ activity in iCAN-DO was logged in the portal. Information about general online activity was retrieved at the time of the interviews.

#### Interviews

The second author (AH) was the interviewer. AH is a registered specialist nurse with experience in palliative cancer care. AH has been involved in some parts of the development and delivery of the stepped-care intervention, but has no overall responsibility in the program. An interview guide with open-ended questions was used, containing questions regarding participants’ user experiences of iCAN-DO. Follow-up questions were based on each informant’s responses and were used to acquire more thorough descriptions of experiences [19]. Questions such as “How did that feel?” or “How did you use that?” were used, as well as probing and interpreting questions. One test interview was performed to explore the design and understanding of questions, but no adjustments to the interview guide were needed. Informants were offered the option to have iCAN-DO open during the interview, if they wanted to. The interviews were performed in a place chosen by the informant; some informants chose their place of work, some wanted to meet in their home, and a few chose the hospital. The interviews were tape-recorded and lasted between 45 and 120 minutes.

### Data Analysis

Interviews were transcribed verbatim by AH, and latent content analysis [20] was used to analyze the interviews. The text was read several times and the manifest content (ie, what is explicit and obvious in the text) of each interview was divided into meaning units and condensed meaning units. Each condensed meaning unit was then given a code to describe the key message, and codes with similar content were allocated to the same category (see Table 2). An interpretation of the latent content (ie, the underlying meaning of the text) was made by abstracting the underlying implicit aspects of the manifest and explicit content. Thus, when several categories were identified as containing similar, repeating ideas, a subtheme was formed. The implicit underlying meaning of all subthemes was then abstracted to one overarching theme. AH and HI were mainly responsible for the analysis, but the group of authors—mainly HI, AH, BJ, and SA—discussed and considered the results on several occasions. The quotes presented in the paper were translated by the first author (HI), AH, and Linnea Holmén at Calyptic.

**Table 1:** Informants’ characteristics at time of inclusion in the randomized controlled trial (RCT) AdultCan.

Characteristic				Value (N=15)
Age (years), mean (SD); min-max				58.9 (8.9); 37-69
**Gender and diagnosis^a^, n (%)**				
	**Female**			
		Total	10 (67)	
		Breast cancer	10 (67)	
	**Male**			
		Total	5 (33)	
		Colorectal cancer	1 (7)	
		Prostate cancer	4 (27)	
**Relationship status, n (%)**				
	Married or partner and living with someone		12 (80)	
	Married or partner but living alone		1 (7)	
	Widowed		1 (7)	
	Single		1 (7)	
**Level of education, n (%)**				
	Elementary school		3 (20)	
	High school		1 (7)	
	University up to 3 years		6 (40)	
	University more than 3 years		5 (33)	
**Working situation, n (%)**				
	Working		10 (67)	
	Retired		4 (27)	
	Early retirement		1 (7)	
**General online activity (outside the portal), n (%)**				
	Daily with no social media		4 (27)	
	Daily and active on social media: lurking		7 (47)	
	Daily and active on social media: participating		4 (27)	
**Activity in Step 1, n (%)**				
	Opening material (all sections^b^) >20 times		6 (40)	
	Opening material (some sections) >20 times		7 (47)	
	Opening material (some sections) <10 times		2 (13)	
**Participation in Step 2 (iCBT^c^), n (%)**				
	No		9 (60)	
	Yes		6 (40)	

^a^Diagnosis percentages are out of the total number of informants (N=15).

^b^Library, peer support, frequently asked questions, or Ask an expert.

^c^iCBT: internet-based cognitive behavioral therapy.

**Table 2:** Steps in the content analysis.

Meaning unit	Condensed meaning unit	Code	Category
That you can sit at home, in peace and quiet, when you feel like it and something comes up. You seldom come to think of things when you have the possibility to ask questions, at the hospital.	Studying the material when the questions arise	Available when the need arises	A web portal provides high availability and accessibility
The disease means that it’s hard for me to get going, I feel best in the evening and that’s when I can gather information in the portal.	Being able to gather information when I feel well	Availability tailored for my health	A web portal provides high availability and accessibility

## Results

### Overview

In the analysis, 10 categories were derived concerning user experiences and perceptions of delivery, design, and structure of iCAN-DO. The categories were abstracted into three subthemes with the overarching main theme *The cancer disease and its treatment place high demands on accessibility and user experience* (see Table 3).

### Subtheme 1: User Experience in the Context of Cancer

The personal situation and consequences of illness, both somatic and cognitive, were mentioned by all informants as factors that in various ways affected their ability to make use of iCAN-DO. Symptoms of fatigue and pain could require being in a comfortable position when listening or reading. Some informants considered it facilitating to be able to use iCAN-DO on tablets as well as on a computer, even though the design was not optimized for mobile devices. Furthermore, having direct access to iCAN-DO was perceived as beneficial by those experiencing a lot of time constraints in everyday life. They could use iCAN-DO when it suited them and when they had the time.

The information in Step 1 was provided in different formats, and informants had varying preferences in regard to the material. Some assimilated the contents best by reading, others by listening to audio clips or watching video clips, and others by using some combination of these formats. Some printed all the material and read it on paper. Though video facilitated immersion in the material, in some cases video clips were considered too long or had poor audio quality, which hampered the informants’ ability to pay attention. Furthermore, informants stated that answering questionnaires evoked both negative and positive feelings, but also made them reflect on their health. They felt questionnaires could be a part of health care, for example, as preparation ahead of a visit to oncology care.

### Subtheme 2: Technical Struggles Require Patience and Troubleshooting

Many of the informants described the multistep log-in as an overly difficult way to log in, and an option of using Mobile BankID, an electronic identification application, would have been preferred by most. Some of the informants described how having different log-in codes caused problems. The multistep log-in also reduced spontaneous visits. The informants also described other technical struggles, such as the need for separate add-ons to make the page work and a perceived instability, for example, being involuntarily logged out and difficulties when communicating with the psychologist. Some of these problems were described as transient and occurring only in the initial phase, while others were seen as persistent. Perceived technical difficulties were described as a factor that caused negative feelings, lack of motivation, and a postponing behavior.

The prevalence of technical problems required a lot of problem solving. The informants described different ways of handling problems. Some informants described solving problems themselves by installing new software or requesting a new password. Others enlisted the help of relatives or friends. The importance of technical support was highlighted and those who contacted information technology (IT) support were satisfied with the help they got. Some preferred calling and others sent an email or direct message via the portal. Technical support was described as crucial for not giving up.

The informants also had suggestions for improvements to the interface. An aspect described as important was to receive a confirmation that a written message had reached the help desk when awaiting an answer.

### Subtheme 3: Appealing and Usable, but Rather Simple

iCAN-DO was experienced as easier to use than many other systems, and getting access to the contents was seen as more important than getting an advanced system. Some informants had the perception of an intuitive interface that was easy to understand and navigate, while others perceived the same simplicity as a shortcoming. The graphical design was described by some as simple and serving its purpose, while others found it dull or overly intricate. The experiences varied between informants who were experienced everyday internet users with high interest in social media and those who were more inexperienced with little or no use of social media.

When participants had questionnaires to answer in the portal, repeated reminders were sent via SMS. Informants described the reminders as a trigger for accessing the supporting material as well, as it meant they logged in. The informants also came up with ideas regarding functions that could improve iCAN-DO, such as better layout of questionnaires, adding news coverage, and adding graphical displays of self-reports.

**Table 3:** The results of qualitative content analysis—subthemes, categories, and quotes—for the theme The cancer disease and its treatment place high demands on accessibility and user experience.

Subtheme and categories		Quotes
**User experience in the context of cancer**		
	My health status and environment affect how I use the portal	“The tablet is light and the computer is heavy...so if my body is hurting in every fiber, I don’t want to have something heavy in my lap, so I used the tablet almost all the time...” [Informant #3]
	Multiple delivery modes enable assimilation of the content	“The mini lectures have been good, maybe they could have been a bit shorter...because it’s been tough when you’re sick to listen for such a long time.” [Informant #13]
	A web portal provides high availability and accessibility	“Like, when you are on a bus or train you might want to use the time to check something, you have to be able to do that. Right now, I use my smartphone more than my computer; I only use the computer when I really have to.” [Informant #7]
	Questionnaires make you reflect on your health	“I could feel a little...well, blue, afterwards. You think...Should I feel like that too?” [Informant #2] “...even just answering the questions has been therapeutic. I noticed a difference myself too. At the start I indicated how bad I felt and I noticed that it did actually get better.” [Informant #12]
**Technical struggles require patience and troubleshooting**		
	Complicated multistep log-in procedure	“It was awfully tricky, and I should really be able to do it, because I’ve worked a bit with VPN [virtual private network] connections, but this one crashed several times for me; it didn’t work...” [Informant #3]
	Annoying technical problems reduce motivation to use the portal	“You get a response within the response itself and then I have an inbox as well, where it ended up. So you have it in two places and even though I have read the response, it still looks unread in the inbox.” [Informant #9] “Technical hassles are not good, it’s very annoying...and once you postpone your visit it might never happen.” [Informant #10]
	Troubleshooting and the importance of support	“Because otherwise, if you don’t get help, you feel like you don’t give a damn...When there are technical difficulties on the site you postpone your visits, I will log in later...but then you never do it. But then at the end I got information [from the help desk] so...” [Informant #1]
**Appealing and usable, but rather simple**		
	Intuitive but unstable interface	“I thought it was easy to see what it was about, what they had...if there was something that appealed to you, then it was easy to access and I could see what it said. I’m not a computer person, really, but I managed pretty well, I think, and that’s a good sign.” [Informant #4]
	Appealing, yet simple graphical design	“For people in general, it’s probably pretty good that it is so simple. Because then it will be used.” [Informant #14] “I am into Instagram, Facebook, and all those places, you know, so I felt this was...a bit boring. I am sorry to say that, but that’s just me. It doesn’t give me the kind of input and variety I need.” [Informant #11]
	Technology that creates motivation and innovation	“It was good to have the reminders, because sometimes you maybe don’t have the energy the first time, you’re having a bad day, but then maybe you can manage it after the second or third reminder...I have often looked around a bit at other things when I was logged in on the portal anyway.” [Informant #11] “In that part, you would want a graphical...like a curve so that you can see it yourself...Another thing that I’ve thought about is that there could be like a news function, things that are going on within oncology care, like in all of Sweden, maybe.” [Informant #12]

## Discussion

### Principal Findings

According to the informants, the user experience of iCAN-DO was affected by physical and cognitive symptoms, as well as time constraints in everyday life, which all highlighted the importance of 24-hour access, a responsive design, and a well-functioning system providing access to technical support.

Both health-related problems and external circumstances may constitute barriers for making contact with health care in the daytime or during working hours. By using iCAN-DO, informants could study information at a time suitable to their situation and health, and could review the information as many times as needed. The delivery of content in different formats also stood out as a positive feature, as informants reported such varied preferences in this regard. In the developmental phase, the use of different information formats was suggested as highly important by patient representatives, who had their own experiences of fatigue or other debilitating symptoms. Further, the participants underlined the benefit of the contents being accessible via different devices, such as computer, tablet, or mobile phone. In the trial, a responsive design was later incorporated into the software in order to adapt the portal to mobile devices, including smartphones and tablets. Regardless of which functions are incorporated and what devices are used, it is important to include functions for recording portal activity and usage, to secure internal validity [21].

Informants also described various aspects of filling out questionnaires. Some reported that the questionnaires reminded them of their situation in a negative way, but others perceived the questionnaires as enlightening and providing them with feedback on their own health over time. Other studies have reported promising results on individual tailoring of content depending on diagnosis, treatment, and even tracking of symptoms [22], given that the individual does not feel distress at such monitoring [23]. To motivate patients and professionals to use patient-reported symptoms, it is important to underline the aim of this use and to clearly state which professionals have the responsibility to review and discuss the results [24].

Various aspects of technical struggles were revealed in the interviews. Before launching iCAN-DO in *AdultCan*, user-experience testing was performed in various settings, by both professionals and patient representatives. Still, informants in this study described difficulties, and technical support was crucial for not giving up. Experiencing difficulties when attempting to use a program has been described to influence perceptions of its value as a whole. However, training and technical support can make users feel more capable over time [25,26]. This is in line with the Technology Acceptance Model [27], which posits the predictive power of the person’s perceived usefulness of the system and ease of use. Though the informants in this study mentioned perceived benefits from the 24/7 access and the multifaceted information, the technical problems when logging in or filling out questionnaires were described as hampering use. Some problems were transitional and could be solved. The possibility to contact a support desk was crucial, suggesting that a highly accessible support function may be very important for new web portals. A take-home message is that electronic health (eHealth) initiatives, to a higher extent, should look into how to provide more user support using technology. For instance, in multiple fields of practice, there is an increasing use of chatbots (ie, text-based conversational interface) to provide customers with support. Such technologies have also been considered in the health care setting when it comes to medical counseling (eg, in oncology care [28]), but could be extended to include technical support to (1) provide a better user experience and (2) increase the scalability of eHealth solutions by reducing the need for support desk staff.

Since iCAN-DO was part of an RCT, an authorization procedure was necessary for control and internal validity. However, many informants perceived the multistep log-in procedure as burdensome, which has also been recognized in similar platforms [29]. After this set of interviews, access to iCAN-DO has been facilitated by the incorporation of Mobile BankID, which drastically reduced the need for support regarding log-in matters. BankID is the leading electronic identification solution in Sweden and has around 8 million active users out of the total population of 10 million people. Even though this particular solution is not available in all countries, we want to stress the need to investigate and adopt electronic identification solutions that are prevalent in the national care provision context and that are well known to the users.

The experiences of the design and the user experience of iCAN-DO varied between the informants who were experienced internet users with a high interest in social media and those with little or no use of social media. As stated earlier, patient representatives from the three diagnostic groups participated in the development of iCAN-DO to increase the relevance of the internet-based stepped care. This group of patient representatives was a quite homogenous group with regard to their opinions about the graphical design, stressing the need for simplicity. It is worth highlighting that more computer experience has been reported to be associated with increased use in another web-based intervention for patients with cancer [30]. In a review of patient web portals for primary and secondary prevention, sociodemographic factors, such as belonging to a racial and ethnic minority, having less education, and having lower health literacy, have been found to be associated with less system use [31]. Thus, when designing future web-based interventions, efforts should be made to do a user analysis and to include patient representatives with varied experience of social media, internet use, education, health literacy, and ethnicity. Furthermore, future web portals not restricted to research projects may benefit from updating the content at regular intervals [28] and incorporating features with news and statistics.

### Methodological Considerations

The aim of this study was to evaluate the user experiences of iCAN-DO used in *AdultCan*; thus, the only people available for inclusion were participants in the ongoing trial, which might lower the transferability to other people and contexts. The sample also had a predominance of female informants with breast cancer, reflecting the total sample in the RCT. The majority of the informants were well educated, which may act as a confounder for both perceived usefulness and user experiences of the portal and its material. Beyond differences in diagnoses and education, informants in this study varied in other aspects, such as general online activity and age. Even though the results relate only to this particular portal and web-based support, the take-home message regarding accessibility, delivery mode, technical matters, and design aspects is one that researchers and designers of other web-based support might benefit from. Future studies should consider the user experience in light of not only age, gender, and diagnosis but also factors such as ethnic background, education, and health literacy.

### Conclusions

The high accessibility and variety of delivery modes provided by iCAN-DO were important, but consequences of illness, as well as individual situations and preferences, placed high demands on user experience. Technical struggles decrease motivation and usage, which highlights the importance of a support function. Users’ internet activity, computer experience, and interactions in social media are aspects that seem to impact on the perception of the interface and graphical design. Future web portals would gain from tailoring both content and design to individual preferences.

## Abbreviations

eHealth: electronic health
FAQ: frequently asked questions
HADS: Hospital Anxiety and Depression Scale
iCBT: internet-based cognitive behavioral therapy
IT: information technology
RCT: randomized controlled trial
U-CARE: Uppsala University Psychosocial Care Program
